# Androgens downregulate miR-21 expression in breast cancer cells underlining the protective role of androgen receptor

**DOI:** 10.18632/oncotarget.7207

**Published:** 2016-02-05

**Authors:** Ivan Casaburi, Maria Grazia Cesario, Ada Donà, Pietro Rizza, Saveria Aquila, Paola Avena, Marilena Lanzino, Michele Pellegrino, Adele Vivacqua, Paola Tucci, Catia Morelli, Sebastiano Andò, Diego Sisci

**Affiliations:** ^1^ Department of Pharmacy and Health and Nutritional Sciences, University of Calabria, Arcavacata di Rende (CS), Italy

**Keywords:** androgen receptor, breast cancer, miR-21, androgens

## Abstract

Although the protective role of androgen receptor (AR) in breast cancer (BC) is well established, the mechanisms involved remains largely unexplored. MicroRNAs play fundamental roles in many biological processes, including tumor cell development and metastasis. Herein, we report that androgens reduce BC cells proliferation acting as a negative modulator of the onco-miRNA-21.

The synthetic androgen miboleron (Mib) decreases BC cell proliferation induced by miR-21 over-expression and AR knockdown evidenced the requirement of AR in the down-regulation of miR-21 expression. These effects seem to be a general mechanism occurring in BC tissues.

Chromatin immune-precipitation (ChIP) analysis disclosed the binding of AR to a specific ARE sequence in miR-21 proximal promoter and recognizes the recruitment of HDAC3 as component for AR-mediated transcriptional repression. Such event is associated to a significantly reduced PolII binding in Mib treated extracts confirming that activated AR is a transcriptional repressor of miR-21 expression, providing further insight into the protective role of androgens in breast cancer cells.

Collectively, our data and the widespread AR expression in primary and metastatic breast tumours, suggest a careful examination of the therapeutic potential of androgens also in potentiating the effectiveness of anti-oestrogen adjuvant therapies.

## INTRODUCTION

The importance of androgens in the treatment of breast cancer (BC) has been reported in many studies, but the role of androgen receptor (AR) remains not completely elucidated. AR is expressed in the majority of primary tumors [1] and in many of the metastatic lesions [2]. Although estrogen receptor α (ER) plays a pivotal role in driving BC growth, AR is the most commonly expressed hormone receptor in “*in situ*”, invasive and metastatic BC. It is well known that BCs are classified based on ER, progesterone receptor and HER2neu expression but, as a consequence of the importance of AR activity a reclassification of BCs into three subtypes based on the expression of ER and AR has been proposed [3]: luminal (ER+, AR+), basal (ER-, AR-) and molecular apocrine (ER-, AR+). AR expression was found to be a favorable prognostic indicator of disease outcome by the majority of studies investigating the relationship between AR levels and the clinical-pathological characteristics in BCs (reviewed in [4]). Moreover, AR content was reported to correlate with a better response to chemotherapy and hormonal therapy [5]. All these effects have been strictly related to ER expression. Recently, the overall survival and the disease-free survival that are directly correlated to AR expression have been reported to be irrespective to ER co-expression [6]. Many “*in vitro”* studies have investigated the clinical significance of AR expression and the effects of androgens on BC cell lines, demonstrating the inhibitory role of AR signaling on BC cells proliferation [7-9]. Some of the mechanisms involved in the inhibition of BC cells proliferation have been already elucidated. Specifically, androgens-activated AR inhibits endogenous cyclin D1 expression [10], and down-regulates C-MYC and K-RAS protein expression by up-regulating the miRNA let-7a [11].

MicroRNAs (miRs) are a class of short non-coding RNA genes that act post-transcriptionally as negative regulators of gene expression. A large body of research shows that animal miRs play fundamental roles in many biological processes, including tumor cell development and metastasis [12]. Many are the miRs regulated by androgens in various tissues, such as miR-32 and others in prostate cancer [13], let-7a in breast cancer [11] and miR-21 in prostate cancer [14] and in hepatocellular carcinoma [15]. Among these, miR-21 is considered a key onco-miRNA in carcinogenesis since its expression is consistently high in a wide range of cancers including BCs [16]. Furthermore, miR-21 is the most abundant in breast tumor tissue as compared to matched normal tissue [17], and its expression is higher in invasive and malignant breast tumors [18]. Several potential miR-21 targets have been identified including some tumor suppressor genes such as phosphatase and tensin homolog (PTEN) [19], tropomyosin 1 (TPM1) [20] and programmed cell death 4 (PDCD4) [21].

Considering the oncogenic action of miR-21 in BC and the ability of androgens-activated-AR to bind directly to miR-21 promoter increasing its expression in prostate cancer [14], we evaluated the expression of miR-21 in response to androgen stimulation in BC cells where androgens exert a protective role [7-9].

Herein we demonstrated that, in response to androgens, AR contributes to the reduction of BC cell growth by inhibiting miR-21 expression through the recruitment of HADAC3 on miR-21 promoter.

## RESULTS

### Mibolerone inhibits miR-21 induced breast cancer cells growth

It is well established that miR-21 expression promotes proliferation and invasiveness of breast cancer cells [16]. The oncogenic potential of miR-21 was also evidenced in other cancer cell types, including prostate cancer cells [14], where its expression has been reported to be clearly induced by androgens [14]. Considering that we, and others, demonstrated the existence of some mechanisms by which androgens inhibit BC cell proliferation [10], we investigated if they are able to inhibit BC cell growth also in response to miR-21 overexpression.

**Figure 1:**
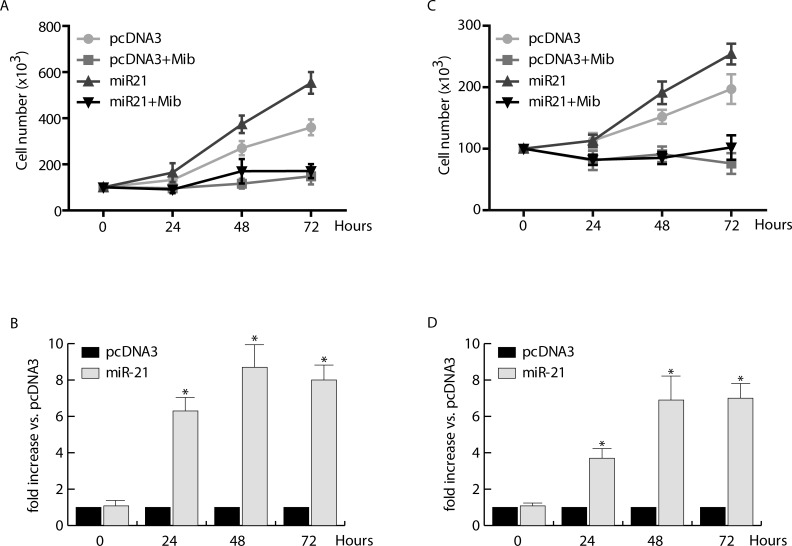
Proliferative effects of miR-21 on human breast cancer cells MCF-7 **A.** and ZR-75-1 **C.** cells were transfected with pcDNA3/pre-miRNA-21 or pcDNA3, allowed to recover overnight, and then incubated in the presence or absence of 10 nM Mib for 24, 48, and 72 hours. Cell proliferation was quantified by trypan blue exclusion. miR-21 expression was evaluated by qRT-PCR on total RNA extracted from transfected MCF-7 **B.** and ZR-75-1 **D.** cells as reported in *materials and methods*. All the qRT-PCR results were normalized to RNU6B and expressed as fold increase *versus* pcDNA3 samples. All the data represent Mean ± SD of three different experiments analyzed in triplicate.

To this aim, MCF-7 cells were transfected with pcDNA3/pre-miRNA-21 and pcDNA3 (control vector) (Figure 1B), synchronized in serum free medium (PRF) for 24 hours (h) and treated with Mib 10 nM in PRF-CT for 24, 48, and 72 h. As expected, Mib inhibited dramatically MCF-7 cell proliferation, while miR-21 overexpression induced about 3 fold increase of cell proliferation (Figure 1A). Interestingly, Mib was able to counteract miR-21 induced MCF-7 cell proliferation. These effects are not related to the cell type but to the tissue since, under the same experimental conditions (Figure 1D), comparable results were obtained in other BC cell lines such as ZR-75-1 (Figure 1C) and SKBR3 (data not shown).

### Mibolerone inhibits basal expression of miR-21 in MCF-7 breast cancer cells

**Figure 2:**
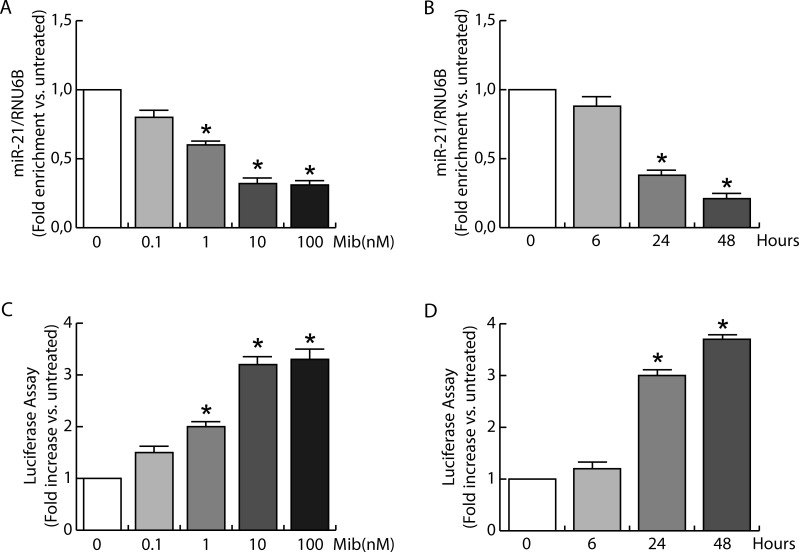
miR-21 expression is inhibited by androgens in breast cancer cells **A.** Total RNA from MCF-7 cells treated with increasing amount of Mib (0, 0.1, 1, 10, and 100 nM) was extracted and miR-21 content was quantified by qRT-PCR. The y-axis represents log fold enrichment after miR-21 pull down, relative to input RNA. **B.** Total RNA from MCF-7 cells treated with Mib (10 nM) was collected after 6, 24, and 48 hours of incubation. miR-21 expression was quantified by qRT-PCR. All the qRT-PCR results were normalized to RNU6B. The regulation of miR-21 expression was evaluated also by transfecting MCF-7 cells with the plasmid Luc-miR-21. MCF-7 cells were treated as reported in A for **C.** and as reported in B for **D.** At the end, cells were subjected to luciferase assay. Renilla tk was used as control of transfection. Data represent fold enrichment with respect to untreated (A, C) or time 0 (B, D) samples, and are reported as Mean ± s.d. derived from three independent measurements *P < 0.05.

Based on proliferation results we questioned if androgens were able to counteract miR-21 action by regulating miR-21 expression in BC cells. To this aim, serum starved MCF-7 cells were left untreated or treated with increasing amount of Mib for 24 h (Figure 2A). The results indicate a significative reduction (60 %) in mature miR-21 content in response to 10 nM Mib. Interestingly, a 10 fold higher concentration of Mib did not exert additional decrease in miR-21 expression.

Since 10 nM Mib were able to strongly reduce the expression of miR-21, we used this concentration to evaluate miR-21 expression in time course experiments (Figure 2B). A marked reduction of miR-21 expression was observed after 24 and 48 h of treatment (60 % and 80 % respectively). These results were also confirmed by evaluating the effect of Mib on miR-21 target gene reporter activity in MCF-7 cells. Starved MCF-7 cells were transfected with pGL3-miR-21-Luciferase and pRL-tk plasmids and then treated with increasing amount of Mib for 24 h (Figure 2C) or subjected to a time course study with 10 nM Mib (Figure 2D). The results revealed an increase of luciferase protein expression, showing that Mib reduces miR-21 expression.

### Androgens reduce miR-21 expression through androgen receptor

**Figure 3:**
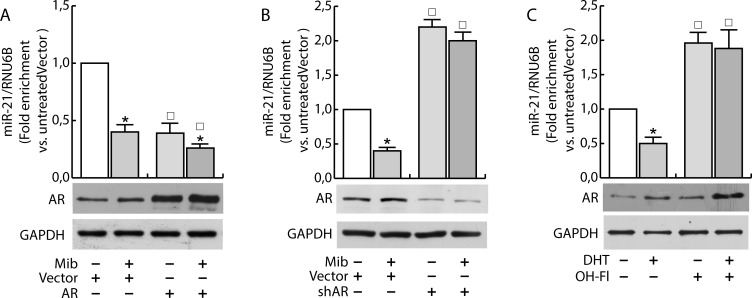
Androgens regulate miR-21 expression in breast cancer cells MCF-7 cells were transfected with Vector (pcDNA3) and the AR expression plasmid (pcDNA2-AR) **A.** or with Vector (psiRNA) and psiAR (shAR) **B.** and treated with Mib (10nM) for 24 h. In addition, synchronized MCF-7 cells were treated with DHT 10 nM and OH-Fl 100 nM for 24 h C). Total RNA was extracted after the treatment and analyzed by qRT-PCR to evaluate miR-21 content. All the qRT-PCR results were normalized to RNU6B. AR expression was determined by western blotting using 30 mg of protein lysates. GAPDH expression was assessed as protein loading control. Data represent fold enrichment with respect to the correspondent untreated Vector sample and are reported as Mean ± s.d. derived from three independent measurements. Statistical analysis was performed applying Student's *t* test: *P < 0.05 is referred to the correspondent untreated sample; □ to the correspondent Vector samples (*p* < 0.05).

**Figure 4:**
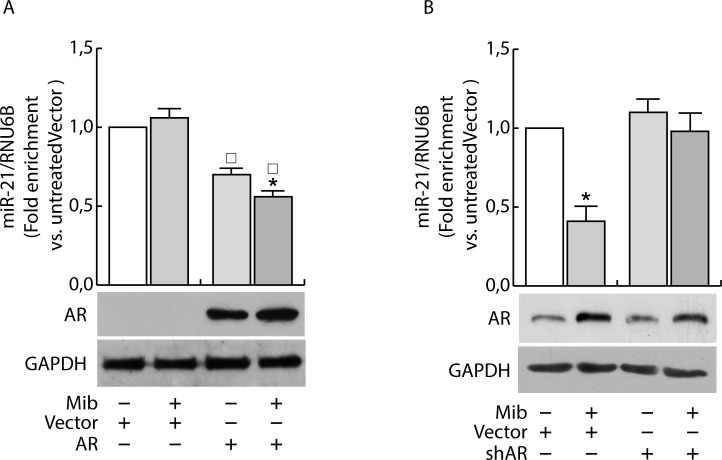
AR regulates miR-21 expression in breast cancer cells MDA-MB-231 cells were transfected with Vector (pcDNA3) and AR expression plasmid (pcDNA3-AR) (AR). After 24 h, cells were treated with Mib (10nM) **A.** for 24 h. ZR-75-1 cells were transfected with psiCon a scrambled shRNA (Vector) or psiAR (shAR) and treated as described before **B.**. At the end of the treatment, total RNA was extracted and analyzed by qRT-PCR to evaluate miR-21 content. All the qRT-PCR results were normalized to RNU6B. Data represent fold enrichment with respect to the correspondent untreated Vector sample and are reported as Mean ± s.d. derived from three independent measurements. Statistical analysis was performed applying Student's t test: *P < 0.05 is referred to the correspondent untreated sample; □ to the correspondent Vector samples (*p* < 0.05).

Having confirmed the ability of androgens to counteract miR-21 induced breast cancer cell growth by reducing miR-21 expression, we evaluated if AR was involved in the down regulation of miR-21. MCF-7 cells were transfected with Vector (pcDNA3) and an AR expression plasmid or with Vector (psiRNA) and psiAR (shAR) to evaluate the involvement of AR overexpression or its knock down on the regulation of miR-21 expression. Treatment with Mib leads to a 50 % reduction of miR-21 expression within the vector transfected group (Figure 3A). Interestingly, AR overexpression was sufficient to reduce miR-21 expression reaching a value comparable to Mib treated vector samples. An additional reduction of miR-21 expression was obtained in AR overexpressing cells exposed to Mib. AR knock-down (Figure 3B) was associated to an increased miR-21 expression even in response to Mib treatment. Overlapping results were observed by treating MCF-7 cells with 5α-Dihydrotestosterone (DHT), an AR natural ligand, indicating that such a reduction was not an artifact of the treatment used. Further, by using Hydroxyflutamide, an AR antagonist (Figure 3C), we confirmed that the inhibitory effect on miR-21 expression is mediated by AR activation (Figure 3C). These results were further strengthened by expressing AR in the AR negative MDA-MB-231 cell line (Figure 4A) or knocking down it in the AR positive ZR-75-1 cells (Figure 4B).

**Figure 5:**
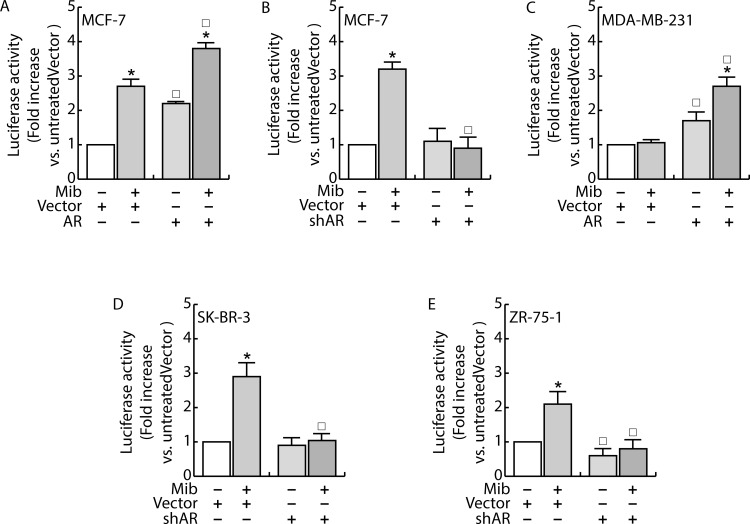
AR regulates miR-21 expression in breast cancer cells MCF-7 **A.** and MDA-MB-231 **C.** cells were transfected with Vector (pcDNA3) and the AR expression plasmid (pcDNA3-AR). MCF-7 **B.**, SKBR3 **D.** and ZR-75 **E.** cells were transfected with Vector (psiRNA) or psiAR (shAR) **B.**. All the cell lines were concomitantly transfected with with Luc-miR-21 and PRL-tk. 24 h after, cells were treated with Mib 10nM for 24 h and, at the end, luciferase content was evaluated as reported in “*Materials and Methods”*. Data represent fold enrichment with respect to the correspondent untreated Vector sample and are reported as Mean ± s.d. derived from three independent measurements. Statistical analysis was performed applying Student's t test: *P < 0.05 is referred to the correspondent untreated sample; □ to the correspondent Vector samples (*p* < 0.05).

Since previous studies have shown that miR-21 is an AR-regulated miRNA in prostate cancer [14] where it promotes cell growth, we questioned if the inhibitory effect observed in MCF-7 is exclusively related to the cell system used or it is a normal response of the mammary epithelium. To exclude the influence of specific factors present in MCF-7 cells, we measured miR-21 expression in other two AR positive breast cancer cells, SK-BR-3 and ZR-75-1, evaluating the ability to repress luciferase expression when the miR is expressed. To this aim, cells were transfected with MiR-21-Luc reporter vector and luciferase expression was evaluated in response to Mib treatment both in Scrambled and shAR cells (Figure 5D and 5E). Also in these cell models, treatment with Mib led to an increase of luciferase activity in Vector transfected cells (Figure 5B, 5D and 5E), that resulted repressed knocking down AR. Moreover, the expression of AR in MDA-MB-231 cell line (Figure 5C) reproduced the same response observed in MCF-7 (Figure 5A). Altogether, these results indicate that AR is required to down regulate miR-21 expression and that it is a general mechanism occurring in breast cancer tissue. In addition, it is also independent from other steroid receptors expression since the effect was observed in MDA-MB-231 cells that are estrogen and progesterone receptor negative.

### Miboleron increased the recruitment of HDAC3 on the miR-21 promoter in MCF-7 cells

To clarify the mechanism through which androgens down-regulate miR-21 expression in breast cancer cells, we verified the binding of AR on the ARE sequence within the miR-21 promoter.

**Figure 6:**
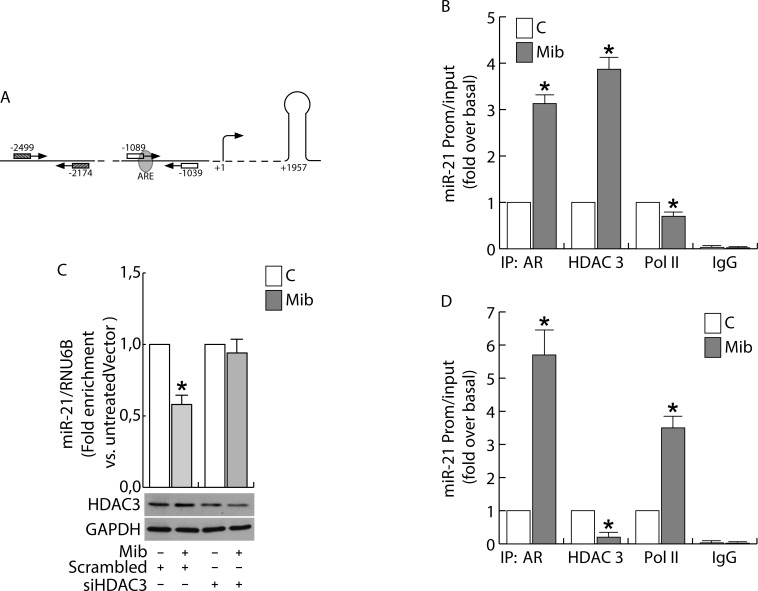
Androgens regulate miR-21 expression by binding to miR-21 promoter in breast cancer cells ChIP analysis was performed on nuclear extracts from sub-confluent MCF-7 **B.** and LNCap **D.** cells, switched to PRF-CT. 24h later cells were left untreated or treated for 45 min with 10 nM Mib. The ARE-containing miR21 promoter region **A.**, was precipitated with either anti-AR, anti-HDAC3 or anti-Pol II Abs and amplified using a specific pair of primers reported in “*Materials and Methods*”. Mib-treated samples were also precipitated with normal rabbit IgG that was used as negative control. In addition, a 325 bp fragment located at −2051/−1726 was amplified on the same precipitated samples as qualitative control. HDAC3 expression was knock down by transfecting siRNA recognizing HDAC3 mRNA (siHDAC3) or a Scrambled siRNA in MCF-7 cells **C.** as reported in *“Materials and Methods*”. After 24 h, cells were treated with Mib (10nM) for 24 h. At the end of the treatment, total RNA was extracted and analyzed by qRT-PCR to evaluate miR-21 content. HDAC3 expression was determined by WB using 30 mg of protein lysates. GAPDH expression was assessed as protein loading control. Data represent the mean ± s.d. of three independent experiments. Statistical analysis was performed applying Student's *t* test: * *p* < 0.05 *vs*. untreated.

Considering that an ARE sequence involved in the direct transcriptional regulation of miR-21 induced by AR binding to the miR-21 promoter has been previously reported by Ribas et al. in prostate [14], we questioned if the same responsive element may be still involved in the negative regulation of miR-21 expression in breast cancer cells (Figure 6A). Chromatin immune-precipitation (ChIP) analysis was performed in nuclear extracts from MCF-7 and LNCap cells. There was no recruitment of AR to the negative control region (see “*Materials and methods*”) and no amplification of PCR products in ChIP reactions using IgG. By contrast, a significant recruitment of AR on the region containing the ARE sequence was observed in response to Mib exposure (Figure 6B). Interestingly, the increased recruitment of AR was not paralleled by PolII binding that resulted significantly reduced in Mib treated extracts (Figure 6B). To explain the different response to androgen stimulation observed in BC cells with respect to prostate cancer cells, we focused our attention to fast mechanisms that may alter the transcriptional response to a stimulus. The same samples were precipitated with HDAC3 whose binding to the promoter resulted strongly increased in response to Mib (Figure 6B). The involvement of HDAC3 in the regulation of miR-21 expression by AR was confirmed by knocking down HDAC3 in BC cells (Figure 6C). Interestingly, HDAC3 is not recruited on miR-21 promoter in LNCap cells in response to Mib treatment (Figure 6D). All together, these results may evidence the involvement of an epigenetic mechanism in determining an opposite response of prostate and BC cells to Mib treatment.

## DISCUSSION

The interest on androgens as factors involved in BC progression was intuited several years ago through the “hyperandrogenic” theory on the basis of which an increased androgenic activity is present in BC [22]. Although androgens were successfully used to treat BC [23, 24], the role of AR and its signaling in breast carcinogenesis are not yet elucidated and are very often controversial [4]. AR expression in BC tissue samples has been associated with a better prognosis [25, 26] and the lack of AR expression correlates with transformation from “*in situ*” to invasive basal subtype ductal breast carcinoma [27]. In addition, loss of AR in triple-negative breast cancers is associated with a worse prognosis, including those with basal-like features [28]. Conversely, some reports suggest that AR may participate to the development of invasive ductal carcinoma by repressing E-cadherin expression [29]. It is worth noting that, most of these results have been obtained stimulating the cells with enzymatically metabolizable androgens, such as testosterone and DHT, while non-metabolizable AR agonists are now used for the treatment of breast cancer. Some of the mechanisms by which AR inhibits breast cancer cell growth have been explained, for example through the down regulation of cyclin D1 [10], by increasing DAX 1 expression that causes an inhibition of aromatase content [30], by up-regulating ER-beta gene expression [31], by down-regulating CMYC and KRAS expression in response to let-7a increased expression [11], and more. Here we demonstrated that AR inhibits breast cancer cell growth by down-regulating miR-21 expression.

The involvement of miR-21 in both carcinogenesis, by increasing the growth of breast cancer cells [16], and tumor progression and invasion [18] is well described. miR-21 expression is regulated in response to various stimuli, including androgens, as demonstrated by Ribas et al. in prostate cancer cells [14]. Interestingly, in prostate cancer cells androgens increase miR-21 expression by trans-activating miR-21 promoter. Our data provide evidence that in breast cancer cells activated AR is a transcriptional repressor of miR-21 expression. Analysis of the molecular events associated with the negative regulation of miR-21 transcription disclose the binding of AR to a specific ARE sequence in miR-21 proximal promoter, and recognizes the recruitment of HDAC3 as component for AR-mediated transcriptional repression. HDACs are well known to repress the transcription of genes regulated by multiple nuclear receptors [32]. In response to receptor antagonists HDACs are recruited at hormone receptor binding sites to block ligand-induced gene expression. Interestingly, AR promotes the recruitment of the repressor complex to the ARE binding site in miR-21 promoter previously indicated by Ribas et al. [14] as the AR permissive binding domain for miR-21 transcription in prostate cancer. These data indicate a specificity in AR functional response depending only by cell type that leads to the protective role of androgens in breast cancer cells. Paradoxically, HDACs are also required for the activation of a fraction of AR target genes of human prostate cancers [33]. These observations clearly indicate the different role played by AR in breast cancer with respect to other cancer, such as prostate [14] and liver [15].

A discrepancy appears considering the suppression of mir-21 expression at promoter level and the inhibition of proliferation induced by ectopic expression of miR-21 in response to androgens (Figure 1). Such a discrepancy may be explained taking into account the ability of androgens to inhibit Cyclin D1 expression in breast cancer cells [10], whose translation is accelerated by miR-21 [34].

Collectively, these data and the widespread expression of AR in primary and metastatic breast tumours, justify a careful examination of the therapeutic potential of androgens also in potentiating the effectiveness of anti-oestrogen adjuvant therapies.

## MATERIALS AND METHODS

### Cell culture, conditions, and treatments

Authenticated human BC epithelial cell lines MCF-7, ZR-75-1, MDA-MB-231, SKBR3 and LNCap were purchased from Interlab Cell Line Collection, Italy. Cells were stored according to supplier's instructions and used within 4 months after frozen aliquots resuscitations (less than 30 passages). Every 4 months, cells were authenticated by short tandem repeat analysis (AmpFLSTR Profiler Plus PCR Amplification Kit, Applied Biosystems) at our Sequencing Core; morphology, doubling times, estrogen sensitivity, and mycoplasma negativity (MycoAlert, Mycoplasma Detection kit, Lonza) were tested.

MCF-7 and ZR-75-1 were maintained in DMEM/Ham F-12 medium (1:1) (DMEM/F-12) supplemented with 5 % Fetal Bovine Serum (FBS). MDA-MB-231 were cultured in 10 % FBS DMEM, LNCaP were grown in RPMI 1640 supplemented with 10 % FBS. Additionally, culture media were supplemented with 100 IU/ml penicillin, 100 ng/ml streptomycin, and 0.2 mM L-glutamine. For experimental purposes, cells were synchronized in phenol red-free (PRF) and serum-free media (SFM) for 24 hours (h) and then switched to PRF-media containing 5% charcoal-treated FBS (PRF-CT) in the presence or absence of Mibolerone (Mib, from Perkin Elmer), 5α-Dihydrotestosterone (DHT, Sigma) and hydroxyflutamide (OH-Fl, from Sigma Aldrich). All media were purchased from Invitrogen, Italy.

### Plasmids and transfections assays

The following plasmids were used: pcDNA3 empty vector (Life technologies); pcDNA3-miR21, for the expression of pre-miR-21 (provided by Joshua Mendell, Addgene plasmid # 21114) [35]; the vector-based pSiAR plasmid (shAR), coding for a small interfering RNA targeting the 5′-untranslated region of AR mRNA, and the scrambled control construct pSiCon (Vector) [36]; pcDNA3-AR (AR) encoding full-length AR [10].

For transfections, MCF-7, SKBR3, ZR-75-1 and MDA-MB-231 were resuspended in PRF-growing medium (PRF-GM) and transfected with Lipofectamine 2000 (Life technologies), according to the manufacturer's instructions. Six hours after transfection cells were synchronized for 24 h and then switched to PRF-CT, in the presence or absence of Mib, for cell growth or RNA extraction purposes.

For luciferase assays, the following constructs were used: Luc-miR-21 [37], a reporter construct for miR activity containing the complementary sequences of mature miR-21 downstream to luciferase, in which Luciferase cDNA was modified in 3′UTR to complementary bind miR-21 (provided by Dr. Qihong Huang) and pRL-Tk (Promega).

### Cell proliferation assays

Cell proliferation was evaluated by cell counting assay as previously described [38]. Briefly, BC cells were seeded on six-well plates (2×10^5^cells/well) in 2.5% PRF-CT. After 24h, cells were exposed to Mib (10 nM) [30] or vehicle for 24, 48, and 72 h. The effects of Mib on cell proliferation were measured at each time point by counting cells using a hemocytometer, and cell viability was determined by Trypan blue dye exclusion test.

### miR extraction and quantitative real-time PCR (qRT-PCR)

Total micro-RNAs were isolated from cells using the *mir*Vana miRNA Isolation Kit (Life technologies) according to the manufacturer's instructions. Total miR were reverse transcribed using a TaqMan MicroRNA Reverse transcription kit (Life technologies) and qRT-PCR was performed with TaqMan universal master mix by using specific primers for miR-21 (all from Life technologies). RNU6B (Life technologies) was used as internal control. Gene and miR expression was defined from the threshold cycle (Ct), and relative expression levels were calculated after normalization to a calibrator that was chosen to be the basal, untreated sample as previously described [39].

### Western blotting assays

Proteins expression was assessed by Western blotting (WB) assay as previously described [40]. Briefly, cells were lysed in Triton lysis buffer, cleared by centrifugation and the protein content was determined by Bradford dye reagent (Bio-Rad). Cellular lysates (20 μg of protein/lane) were resolved by SDS-PAGE, transferred to nitrocellulose membranes and probed with specific polyclonal (p) or monoclonal (m) antibodies (Abs), recognized by peroxidase-coupled secondary Abs, and developed using the Amersham ECL start Western Blotting Detection Reagent (GE Healthcare).

The following Abs were used: anti-AR mAb (441), anti-GAPDH pAb (FL-335) and normal mouse immunoglobulin G (Ig) (all from Santa Cruz Biotechnology). Images were acquired by using an Epson Perfection scanner (Epson).

### Chromatin immunoprecipitation (ChIP)

ChIP assay was performed as previously described [39]. Chromatin extracts were precipitated with anti-AR mAb (441), anti HDAC III (H-99) and anti-Polymerase II (N-20) pAbs (all from Santa Cruz Biotechnology). Normal rabbit IgG (Santa Cruz Biotechnology) was used instead of primary Abs as negative control. Immuno-precipitated DNA was analyzed by qRT-PCR, and the miR-21 promoter sequence containing the Androgen Responsive Element (ARE) was amplified using the following pairs of primers: forward 5′-TCCCAATCATCTCAGAACAAGCT-3′ and reverse 5′- TGCACAGAAACTCCAGTACATTAGTAAC-3′ (50 bp) (Figure 5A) [14]. A sequence upstream of the considered ARE was amplified as control (Figure 5A) forward 5′-CCAGAAGTTAGGGATATGTTAGCA-3′ and reverse 5′-TACCTCCAGGGTTCAAGTGATTCT-3′ (325 bp). Data were normalized with respect to unprocessed lysates (input DNA). Input DNA quantification was performed by using 5 μl of diluted (1/50) template DNA. The relative antibody-bound fractions were normalized as described in “miR extraction and quantitative real-time PCR (qRT-PCR)”. The results were expressed as fold differences with respect to the relative inputs.

### Luciferase assays

MCF-7 were seeded in culture medium on 24-well plates, serum starved for 24 h, co-transfected in PRF-CT with Luc-miR-21 and pRL-Tk, in the presence of pcDNA3, pcDNA3-AR, pSiCon and pSiAR vectors. After 6 h, Mib (10 nM) was added to the medium, where opportune, and the next day cells were harvested, and luciferase activity was measured using dual luciferase assay System (Promega), normalized to renilla luciferase activity (pRL-Tk) and expressed as relative luciferase units.

### HDAC3 silencing

MCF-7 cells were transfected with RNA duplex of stealth siRNA targeted for human HDAC3 (SI03057901), or with AllStars Negative Control siRNA (SI03650318) (both from Qiagen, Milan, Italy). Cells were transfected using RNAiFect Transfection Reagent (Qiagen) as recommended by the manufacturer with minor modifications. After 5 h the transfection medium was changed with SFM, and then the cells were exposed to treatments.

### Statistical analysis

All data were expressed as the mean ± S.D. of at least 3 independent experiments. Statistical significances were evaluated using Student's *t-*test. Statistical significance was accepted as p≤0.05.
